# Dengue Virus Type 2 Infections of *Aedes aegypti* Are Modulated by the Mosquito's RNA Interference Pathway

**DOI:** 10.1371/journal.ppat.1000299

**Published:** 2009-02-13

**Authors:** Irma Sánchez-Vargas, Jaclyn C. Scott, B. Katherine Poole-Smith, Alexander W. E. Franz, Valérie Barbosa-Solomieu, Jeffrey Wilusz, Ken E. Olson, Carol D. Blair

**Affiliations:** Arthropod-borne and Infectious Diseases Laboratory, Department of Microbiology, Immunology and Pathology, Colorado State University, Fort Collins, Colorado, United States of America; The Rockefeller University, United States of America

## Abstract

A number of studies have shown that both innate and adaptive immune defense mechanisms greatly influence the course of human dengue virus (DENV) infections, but little is known about the innate immune response of the mosquito vector *Aedes aegypti* to arbovirus infection. We present evidence here that a major component of the mosquito innate immune response, RNA interference (RNAi), is an important modulator of mosquito infections. The RNAi response is triggered by double-stranded RNA (dsRNA), which occurs in the cytoplasm as a result of positive-sense RNA virus infection, leading to production of small interfering RNAs (siRNAs). These siRNAs are instrumental in degradation of viral mRNA with sequence homology to the dsRNA trigger and thereby inhibition of virus replication. We show that although dengue virus type 2 (DENV2) infection of *Ae. aegypti* cultured cells and oral infection of adult mosquitoes generated dsRNA and production of DENV2-specific siRNAs, virus replication and release of infectious virus persisted, suggesting viral circumvention of RNAi. We also show that DENV2 does not completely evade RNAi, since impairing the pathway by silencing expression of *dcr2*, *r2d2*, or *ago2*, genes encoding important sensor and effector proteins in the RNAi pathway, increased virus replication in the vector and decreased the extrinsic incubation period required for virus transmission. Our findings indicate a major role for RNAi as a determinant of DENV transmission by *Ae. aegypti*.

## Author Summary

Dengue viruses, globally the most prevalent arboviruses, are transmitted to humans by persistently infected *Aedes aegypti* mosquitoes. Understanding the mechanisms mosquitoes use to modulate infections by these agents of serious human diseases should give us critical insights into virus–vector interactions leading to transmission. RNA interference (RNAi) is an innate defense mechanism used by invertebrates to inhibit RNA virus infections; however, little is known about the antiviral role of RNAi in mosquitoes. RNAi is triggered by double-stranded RNA, leading to degradation of RNA with sequence homology to the dsRNA trigger. We show that dengue virus type 2 (DENV2) infection of *Ae. aegypti* by the natural route generates dsRNA and DENV2-specific small interfering RNAs, hallmarks of the RNAi response; nevertheless, persistent infection of mosquitoes occurs, suggesting that DENV2 circumvents RNAi. We also show that DENV2 infection is modulated by RNAi, since impairment by silencing expression of genes encoding important sensor and effector proteins in the RNAi pathway increases virus replication in the vector and decreases the incubation period before virus transmission. Our findings indicate a significant role for RNAi in determining the mosquito vector's potential for transmitting human diseases.

## Introduction

Dengue virus serotypes 1–4 (DENV1-4; *Flavivirus*; *Flaviviridae*) are medically important, positive-sense RNA viruses transmitted to humans by *Aedes aegypti* mosquitoes during epidemic outbreaks [1],[2]. DEN fever and DEN hemorrhagic fever are major public health burdens in many parts of the world [3]; however, although DENVs can cause severe disease in humans, mosquito infections are non-pathogenic and persistent. We hypothesize that the difference in infection outcomes results from host defense (immune) responses. *Ae. aegypti* is an important vector because it feeds almost exclusively on humans and is well adapted to life in tropical urban environments [4]. We have only a rudimentary understanding of DENV molecular interactions with *Ae. aegypti* vectors, including the mosquito's innate defense pathways against arboviruses. DENVs infect the mosquito midgut following ingestion of a viremic blood meal from an acutely infected human, replicate, disseminate to the salivary glands where they are further amplified, and emerge into saliva at the time of transmission. Approximately 10 to 14 days are required for the extrinsic incubation period (EIP), the time between initial infection of the mosquito and transmission [5]. The recent release of the *Ae. aegypti* genome sequence [6] provides an important tool to begin understanding critical virus-vector interactions during the EIP. Identification of mosquito genes that are orthologs of genes known to be part of innate immune pathways in *Drosophila* [7],[8] is an important step in characterizing mosquito defense mechanisms and makes it possible to manipulate putative antiviral pathways during virus infection. Xi et al [8] have recently shown that the *Ae. aegypti* Toll pathway, which is also implicated in *Drosophila* defense against certain viruses, has a role in controlling DENV replication after establishment of a persistent infection.

Recent studies with *Drosophila* clearly show that RNA interference (RNAi) is a potent innate antiviral pathway that is presumably triggered by dsRNA formed in virus-infected cells and leads to degradation of the RNA virus genome. Several groups have shown that RNAi can inhibit infection of *Drosophila* with RNA viruses from the *Dicistroviridae*, *Nodaviridae*, and *Togaviridae* families [9]–[11]. Mutant *Drosophila* lacking functional key RNAi pathway genes such as *dcr2* or *ago2* are highly susceptible to some RNA virus infections [9]–[11]. In *Drosophila*, *dcr2* encodes the RNAi sensor protein Dicer-2 (Dcr2) that recognizes and cleaves long dsRNA, producing 21–25 bp short interfering RNAs (siRNAs) [12],[13]. siRNAs are duplexes with 3′ overhangs of 2 nucleotides and 5′ phosphate and 3′ hydroxyl ends [14]. With the assistance of Dcr2 and the protein R2D2, one strand of siRNA is incorporated into a nuclease complex called the RNA-induced silencing complex (RISC), to start the effector phase of the pathway [15]–[18]. The siRNA strand associated with RISC acts as a guide sequence and anneals to target RNA having sequence complementarity (identity with one strand of the dsRNA trigger) [17],[19],[20]. The Argonaute-2 (Ago2) protein in RISC has sequence specific “slicer” activity and cleaves the target RNA, leading to its degradation [21],[22].

*In silico* searches using sequences of RNAi pathway genes from *Drosophila* show a number of putative RNAi gene orthologs in *Anopheles gambiae* and *Ae. aegypti* genome databases including *dcr2*, *r2d2* and *ago2*. We have carried out limited functional assays to confirm the role of these genes in RNAi [23]–[27]. We have demonstrated that Ago2 expression is essential in mosquitoes for modulation of alphavirus (*Togaviridae*) infection of *An. gambiae* and *Ae. aegypti* [25],[27]. We also have demonstrated that RNAi that inhibits the replication of DENV can be triggered in the midgut of transgenic mosquitoes by expression of an inverted repeat RNA (IR-RNA or dsRNA) derived from a portion of the DENV genome prior to or at the same time as virus challenge in an infectious blood-meal [23]. The Carb 77 transgenic line of mosquitoes expressing DENV2-specific siRNAs generated from the trigger sequence failed to accumulate viral genomic RNA, and poorly disseminated and transmitted DENV2. Significantly, injection of dsRNA derived from either *Ae. aegypti ago2* or *dcr2* sequences prior to ingestion of an infectious blood-meal reversed the resistance phenotype [23]; (Franz and Olson, unpublished data). These studies demonstrated that the RNAi pathway is functional in the mosquito midgut if artificially/exogenously triggered. However, during natural, persistent infections of DENV2 competent mosquitoes, viral RNA is readily detected in midguts from 2–3 days post infection (dpi) to 14 days dpi [5],[28], suggesting that RNAi is not totally effective in preventing replication. An obvious viral strategy to circumvent RNAi is to encode a suppressor of RNAi and a number of examples of suppressors have been described in plant and insect RNA viruses [29],[30], although to date no RNAi suppressors have been associated with arboviruses. Alternatively, DENVs may simply evade RNAi by sequestering their dsRNA replicative forms within virus-induced double membrane structures associated with the endoplasmic reticulum, preventing dsRNA recognition by an RNAi sensor [31],[32]. A focused study of DENV2 infection of competent *Ae. aegypti* should reveal what role RNAi plays in this natural vector-virus interaction. In this paper, we show that persistent DENV2 infections of *Ae. aegypti* cells and mosquitoes generate dsRNA triggers and small DENV2-specific RNAs consistent in size and sequence with siRNAs. Impairment of the RNAi pathway increases the titer of infectious virus in the vector and shortens the EIP, which could increase transmission efficiency.

## Results

### DENV2 growth in *Ae. aegypti* mosquito cells

**Figure 1:**
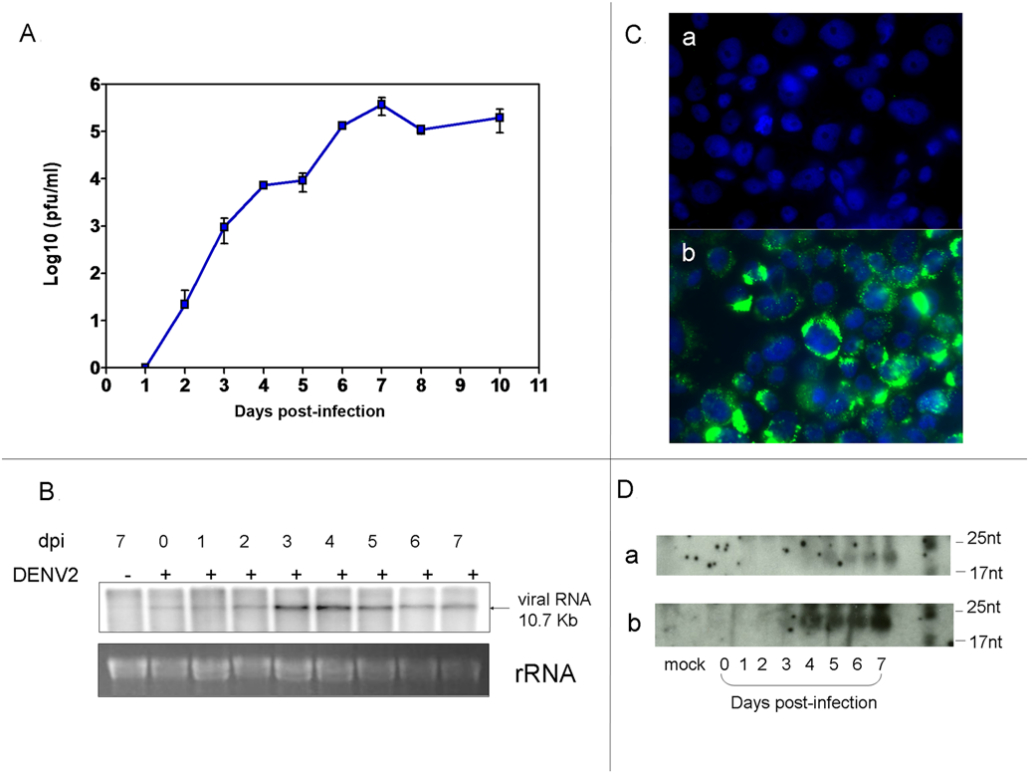
DENV2 infection of Aag2 cells. (A) Growth curve of DENV2 in Aag2 cells. Virus titers in cell culture medium were determined by plaque assay. (B) Detection of DENV2 genomic RNA in Aag2 cells by northern blot analysis (5 µg total RNA/lane). Stained ribosomal RNA is shown as loading control. (C) Detection of dsRNA in mock infected (a) and DENV2 infected (b) Aag2 cells using the dsRNA-specific J2 antibody in an indirect immunofluorescence assay (IFA). (D) Detection of DENV2 genome-derived small RNAs consistent in size with siRNAs on successive days after infection. Small RNAs were detected using sense (a) and antisense (b) riboprobes from the prM gene of DENV2.

We initially used cultured *Ae. aegypti* (Aag2) cells to examine RNAi activity resulting from DENV infection. Triplicate flasks of confluent Aag2 cells were infected at a multiplicity of infection (MOI) of 0.01 with DENV2 (Jamaica 1409, American-Asian genotype) and cell culture medium was collected daily for titration of infectious virus. Virus titer increased daily and peaked at 7 days post infection (dpi) at 6.5×10^5^±2.3×10^5^ pfu/ml (Fig. 1A). DENV2 genomic RNA was detected in cells infected at a MOI of 0.005 by northern blot analysis at relatively high levels by 3 dpi and continuing to at least 7 dpi (Fig. 1B). To determine if the infected cells contained dsRNA, the RNAi trigger, we used the dsRNA-specific J2 antibody [33] in an immunofluorescence assay (IFA) at 3 dpi. Cells that had been mock infected displayed no fluorescence (Fig.1C.a), but strong signal was detected in the cytoplasm of infected cells (Fig. 1C.b), presumably at sites associated with viral replication. We looked for siRNA production by northern blot analysis using total RNA isolated from Aag2 cells. Small RNAs approximately 21 nt in length were detected with both genome-sense (Fig. 1D.a) and antisense (Fig 1D.b) DENV2 prM gene probes beginning at 3 dpi and continuing to 7 dpi. We carried out limited cloning and sequencing of small RNA from Aag2 cells to verify the presence of DENV2 genome sequences and detected a putative siRNA derived from the positive-sense strand of the prM gene (nt 714–733; agtggcactcgttccacatg). Several groups of overlapping 18–22 nt siRNAs appeared to be derived from genome “hot spots”; for example, a total of 9 putative siRNAs were from the nt 4659–5396 region of the NS3 gene, 5 from the positive strand and 4 from the negative strand. Three of these formed an overlapping cluster at nt 4962–4995 (J. Scott and J. Wilusz, unpublished results). The most intense hybridization signal came from the negative sense probe, suggesting that the majority of small RNAs were of the same polarity as genomic RNA.

### DENV2 growth in *Ae. aegypti*

**Figure 2:**
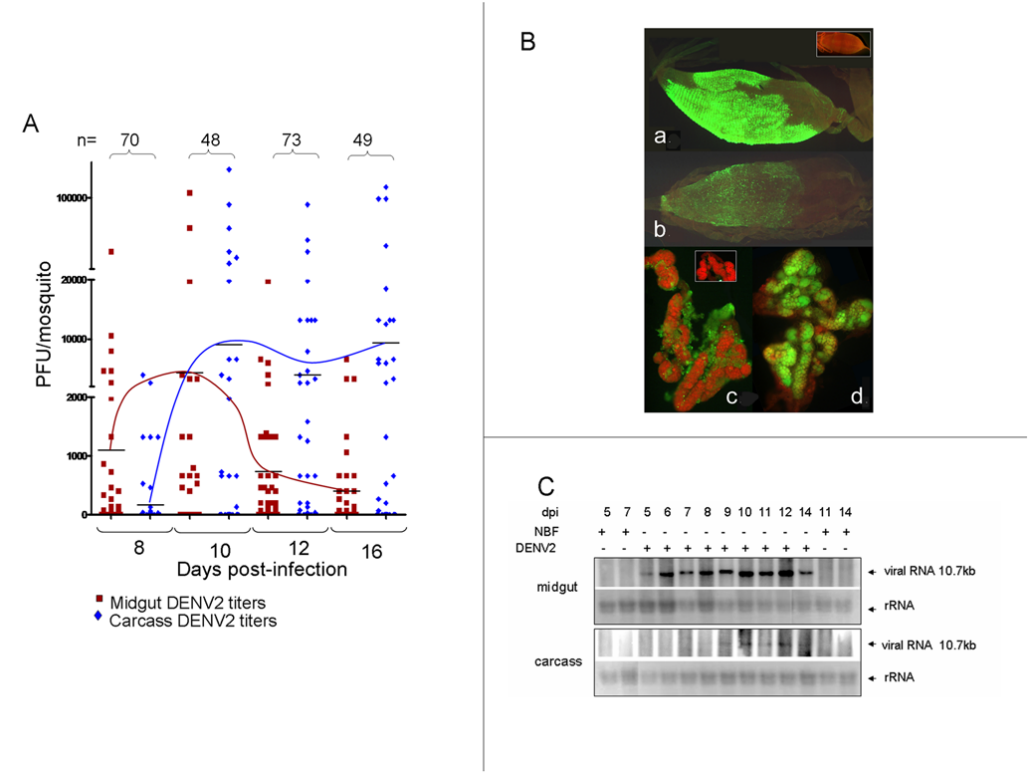
DENV2 infection of *Aedes aegypti* HWE mosquitoes. (A) DENV2 titers in midguts (red symbols/line) and remaining tissues (carcass; blue symbols/line) of individual mosquitoes at pre-determined days post-infection (dpi). (B) Detection of DENV2 E antigen by indirect IFA in midgut (a and b) and salivary gland (c and d) tissue at 7 (a and c) and 14 (b and d) dpi. Insets in panels a and c show IFAs of midguts and salivary glands from non-infected mosquitoes. (C) Northern blot hybridization to determine accumulation of DENV2 genomic RNA in midguts and carcasses from 5 to 14 days post-infection (5 µg total RNA/lane). Ethidium bromide-stained rRNA shown as loading controls. NBF = non-bloodfed (non-infected).

Extensive spatial and temporal analyses were performed to understand the DENV2 infection pattern in our model Higgs white-eye (HWE) Rexville D strain of *Ae. aegypti* mosquitoes following ingestion of an infectious artificial blood meal containing approximately 10^7^ pfu/ml of DENV2. Landmark events in the infection pattern of *Ae. aegypti* HWE mosquitoes are summarized in Figure 2. Infectious virus titers followed a similar pattern as described previously using the same virus strain and various *Ae. aegypti* strains [5],[34] (Fig. 2A). Virus titers increased in the midgut beginning at 3–5 dpi, peaked at 10 dpi (9×10^3^±3.6×10^3^ pfu/ml), and began to decline by 12 dpi (7.4×10^2^±2.9×10^2^ pfu/ml). For HWE mosquitoes, DENV2 envelope (E) antigen was readily detected by IFA in the midgut at 7 and 14 dpi (Fig. 2B.a; Fig. 2B.b), virus dissemination to fat body was detected at about 7 dpi (Fig. 2B.c), and antigen was found abundantly in salivary glands at 14 dpi (Fig. 2B.d). Viral E antigen and virus titer declined in the midgut by 14 dpi but increased in the salivary glands from 14–21 dpi (9.4×10^4^±3.6×10^4^ pfu/ml virus titer in carcass at 16 dpi). Viral RNA could be detected from 5 to 14 dpi by northern blot analysis of total RNA from midguts (Figure 2C). Analysis of remaining tissues (carcass) showed that viral RNA could be detected outside the midgut from 9 to 14 dpi, when the experiment was terminated. In previous studies we have shown that by allowing infected mosquitoes to probe an artificial feeding membrane, we can most consistently detect DENV2 in saliva of HWE mosquitoes at 14 dpi [23].

### Detection of dsRNA and siRNAs in mosquitoes following DENV2 infection

**Figure 3:**
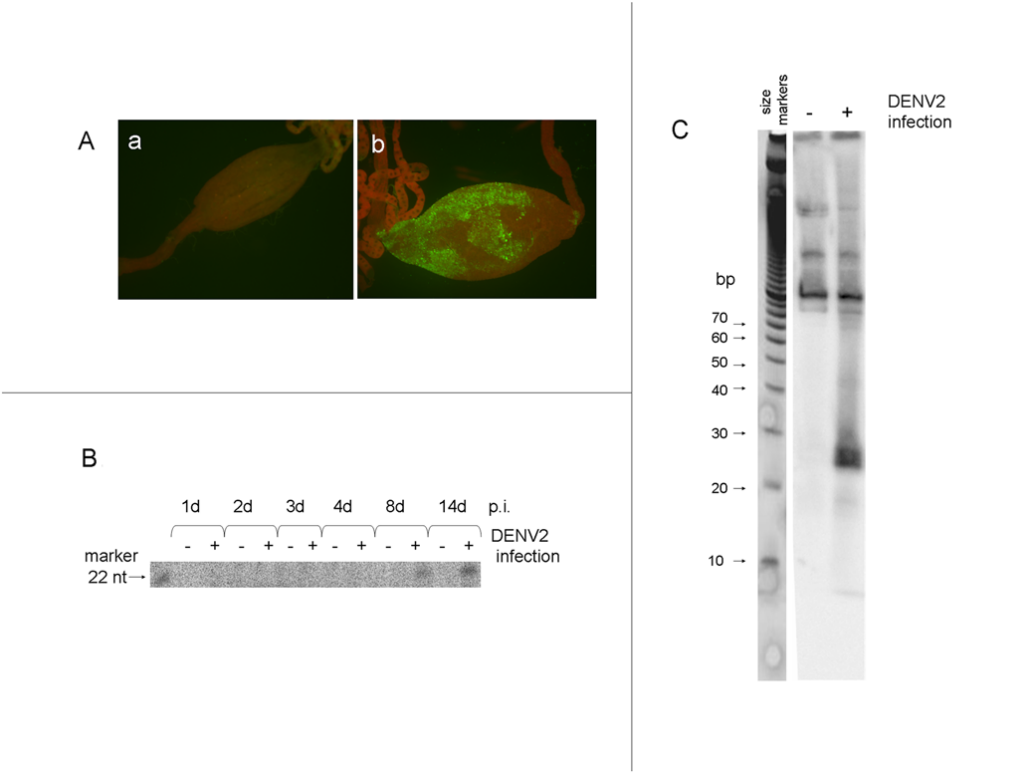
Detection of dsRNA and DENV2-derived small RNAs in infected HWE mosquitoes. (A) Detection of dsRNA in midguts of mock infected (a) and DENV2 infected (b) *Ae. aegypti* using the dsRNA-specific J2 antibody in indirect IFA. (B) Detection of small RNAs in total RNA from DENV2 infected mosquito midguts at various time-points after infection by hybridization with a ^32^P-labeled 22 nt RNA probe complementary (antisense) to the DENV2 prM gene in an RNase protection assay [23]. (C) Northern blot analysis of small RNAs in whole mosquitoes 14 days post infection with DENV2 using a ^32^P-labeled antisense RNA probe from a 298 nt region of the DENV2 prM gene.

The J2 antibody was used to detect dsRNA in midguts, the first mosquito tissue infected after acquisition of a DENV2-containing blood meal. Midguts from mosquitoes that received a blood meal with no virus showed no fluorescent signal (Fig. 3A.a); however, midguts from mosquitoes that had ingested the virus in a blood meal 7 days earlier (Fig. 3A.b) produced a signal reminiscent of DENV2 infection patterns seen by IFA for viral E antigen at the same time point (Fig. 2B.a; [5]. This indicated that DENV2 infection of mosquito tissues also generates the RNAi trigger.

To detect virus-specific siRNAs in infected midguts, total RNA was enriched for small RNAs as previously described [23]. An antisense RNA oligonucleotide from the prM gene that had been previously characterized by cloning and sequencing siRNA was used as an RNase protection probe to detect DENV RNA-derived siRNA in infected midguts [23]. We detected 22 nt RNA at 8 and 14 dpi (Figure 3B). To further verify the existence of siRNAs in mosquitoes following infection, we performed northern blot analysis on total mosquito RNA extracted 14 days after DENV2 infection using an antisense RNA prM probe and detected viral-specific RNAs 22–24 nt in size (Fig. 3C). Thus, in both infected mosquitoes (Fig. 2C and 3B and C) and cultured cells (Fig. 1B and 1D) we demonstrated that viral genomic RNA accumulated concurrently with increasing levels of virus-specific small RNAs, suggesting that the virus employs a mechanism to circumvent the RNAi defense.

### DENV2 growth in mosquitoes after impairment of the RNAi pathway

**Figure 4:**
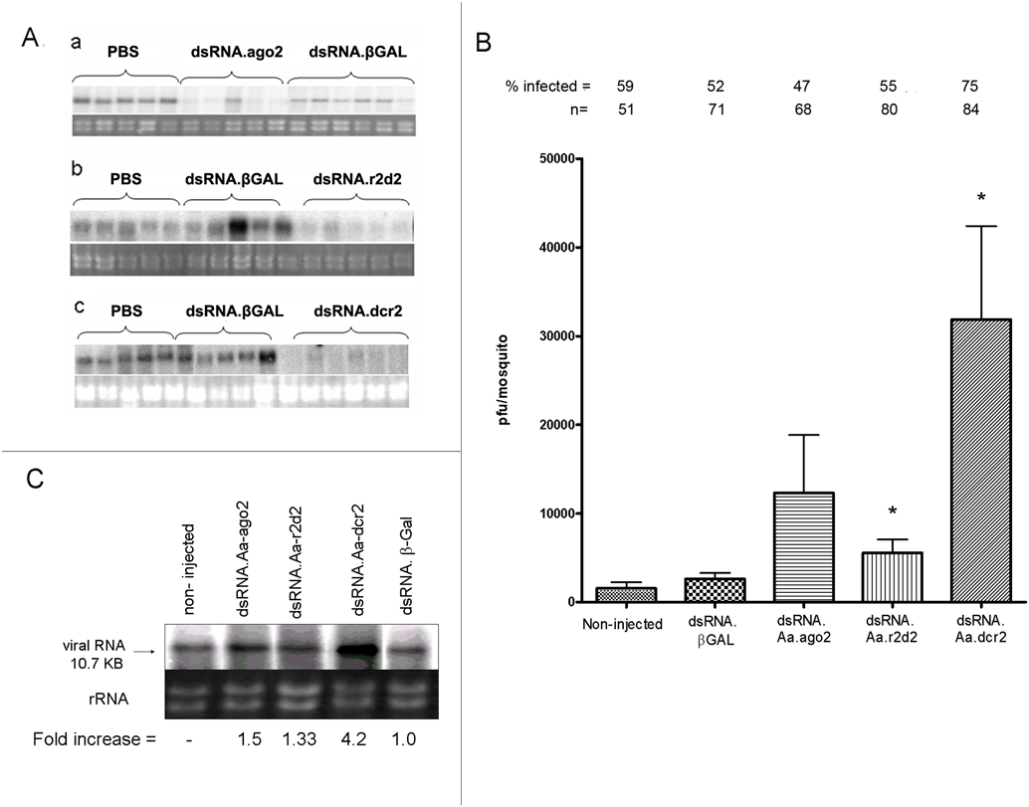
Effects of silencing expression of key RNAi genes prior to DENV2 infection. (A) Northern blot analysis showing RNA silencing of RNAi component genes in individual mosquitoes (3 µg total RNA/lane). Blots were hybridized with antisense ^32^P-dCTP-labeled DNA probes amplified from *Aa-ago2* (a), *Aa-r2d2* (b) and *Aa-dcr2* (c) genes. Ribosomal RNAs are shown as loading controls. (B) Mean DENV2 titers 7 dpi in individual *Ae. aegypti* with an impaired RNAi pathway. Mosquitoes were either non-injected or injected with 500 ng of dsRNA.βGAL, dsRNA.ago2, dsRNA.r2d2, or dsRNA.dcr2 and allowed to recover for 2 days, then given a blood meal containing ∼10^7^ pfu/ml of DENV2. (Bars indicate mean values of titers ±SEM, * indicate P<0.05). (C) Detection of DENV2 genomic RNA by northern blot analysis in whole *Ae. aegypti* at 14 days after virus challenge. Approximately 5 µg of the total RNA extracted from 60 mosquitoes were loaded in each lane. Blots were hybridized with a ^32^P-labeled cDNA probe derived from the prM sequence of DENV2 RNA. Ribosomal RNAs are shown as loading controls. Quantitation of RNA signal was performed using a phosphorimager.

We and other investigators have demonstrated that it is possible to transiently silence (or impair) expression of key components of the RNAi pathway by RNAi knock-down [23]–[25],[29]. We found that injection of dsRNAs derived from RNAi pathway genes into Carb77 transgenic mosquitoes caused impairment of antiviral RNAi, reversing the DENV2-resistant phenotype [23]. To determine if modulation of the RNAi pathway in normal mosquito vectors had an effect on virus replication we injected dsRNA to knock-down expression of key pathway components *Aa-dcr2*, *Aa-r2d2*, and *Aa-ago2*. Primers targeting specific regions of each mRNA and used to generate dsRNAs are listed in Table 1. We intrathoracically injected groups of 200 HWE mosquitoes with 500 ng of dsRNA derived from *Aa-dcr2*, *Aa-r2d2*, *Aa-ago2*, *E. coli βgal* or with PBS. Five mosquitoes from each group were analyzed by northern blot at 2 days after injection to demonstrate silencing of *Aa-ago2* mRNA with dsRNA.ago2 (Fig 4A.a), *Aa-r2d2* mRNA with dsRNA.r2d2, (Fig. 4A.b), and *Aa-dcr2* mRNA with dsRNA.dcr2 (Fig.4A.c) at the time of oral infection with DENV2, which is most crucial in innate defense. Injection of dsRNA.βGAL slightly enhanced, in the cases of *Aa*-*r2d2* and *Aa-dcr2*, and slightly reduced, in the case of *Aa-ago2*, but did not silence any of the RNAi component mRNAs. Although normal levels of mRNA were detectable in some mosquitoes after injection of dsRNA.ago2, injection of dsRNA.dcr2 and dsRNA.r2d2 caused almost complete silencing of cognate mRNA in all mosquitoes tested. To assess the effect of silencing RNAi pathway genes on DENV2 replication, dsRNA-injected mosquitoes were allowed to recover for 2 days, then given an infectious blood meal. Mosquitoes were assayed for infectious virus 7 days later (Fig 4B). The titers in both groups of control mosquitoes, non-injected and injected with dsRNA.βGAL (1.6×10^3^±7×10^2^ pfu/ml and 2.7×10^3^±6.5×10^2^ pfu/ml, respectively) were similar, as were the percentages of mosquitoes infected [59% (30/51), and 52% (36/71), respectively]. In mosquitoes injected with dsRNA.ago2 the titer (1.2×10^4^±6.5×10^3^ pfu/ml) and the number of mosquitoes infected (47%; 36/68) was similar to the non-injected group. In contrast, in mosquitoes injected with dsRNA.r2d2 the infectious virus titer was higher (7.0×10^3^±1.5×10^3^ pfu/ml; P<0.05) than in the non-injected group; however, the percentage of mosquitoes infected (55%; 44/80) was not different from the non-injected group. The most profound effect on DENV2 infection was observed in mosquitoes injected with dsRNA.dcr2 where both the infectious titer (2.5×10^4^±1.0×10^4^ pfu/ml) and the proportion of mosquitoes infected (75%; 63/84) were higher (P<0.05) than the non-injected group.

**Table 1:** Primers used for the generation of dsRNAs targeting RNAi pathway genes.

Gene	GenBank* or Vectorbase ID no.	Sequence, 5′-3′	Target nucleotides	Region
*Aa-dcr2*	AY713296*	Forward GCATTGACGACGAAATCATCGTCCGATG	3758–4250	RNA binding domain
		Reverse ACCATGGCATCCGCCGGTGTCTTGTCC		
*Aa-r2d2*	AAEL011753	Forward ATGGCCTCAAAGCCAGTCCTGAGCAC	76–575	RNA binding domain
		Reverse TTGCAGCGTTCTTAATCATCTCGTTGCACG		
*Aa-ago2*	*Ae. aegypti* Contig_5339	Forward ATGTAGACGCGTCCTCTGT	2380–2880	PIWI domain
		Reverse ACAGTTCAAGCAGACGAACC		

T7 promoter (not shown) was added to the 5′ end of each primer for in vitro transcription of dsRNA.

DENV2 genomic RNA in whole mosquitoes was examined by northern blot analysis 16 days after injection with dsRNA and 14 days after virus challenge. In general, we observed a slight increase in levels of viral RNA in mosquitoes injected with dsRNA.ago2 and dsRNA.r2d2 as compared to control mosquitoes (Fig. 4C). A greater than 4-fold increase in DENV2 genomic RNA signal over controls was seen in mosquitoes injected with dsRNA.dcr2, suggesting that impairment of RNAi early in infection leads to more robust replication of viral RNA in the mosquito.

### Effect of impairment of RNAi on transmission of DENV2 in *Ae. aegypti*

**Figure 5:**
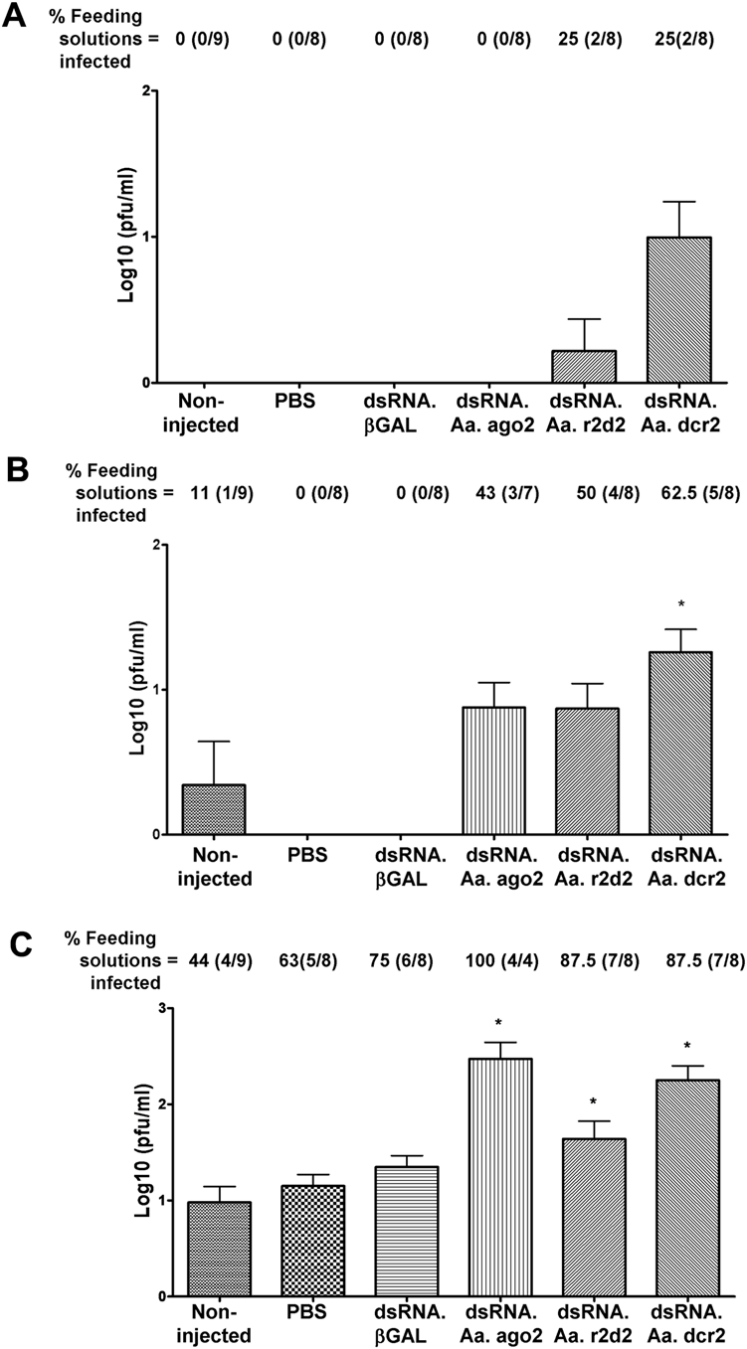
Transmission of DENV2 by *Ae. aegypti* at 7, 10, and 12 days post infection. Groups of 200 mosquitoes were non-injected or injected with PBS or 500 ng dsRNA.βGAL, dsRNA.ago2, dsRNA.r2d2, or dsRNA.dcr2. Two days later all mosquito groups were orally infected with DENV2. At 7 (A), 10 (B) and 12 (C) dpi multiple batches of 10 mosquitoes were allowed to probe artificial feeding solutions. The feeding solutions were assayed for virus titer (* indicate P<0.05).

To determine if transient impairment of RNAi pathway gene expression affected the length of the extrinsic incubation period (EIP) of DENV2 in *Ae. aegypti* HWE mosquitoes we injected groups of 200 mosquitoes with dsRNA.ago2, dsRNA.r2d2, dsRNA.dcr2, dsRNA.βGAL, or PBS and included one non-injected group. Two days later all mosquito groups received an infectious blood meal. Mosquitoes from each group that were engorged with blood were placed into cartons (10 mosquitoes/carton). At 7, 10, and 12 days after infection, mosquitoes in four cartons from each group were allowed to probe an artificial feeding solution for collection of saliva. The feeding solutions were assayed for infectious virus and their mean titers are shown in Figure 5A–C. Virus titers in individual mosquitoes were also determined (data not shown) and indicated that all mosquitoes were infected; therefore lack of virus titer in the feeding solutions was not due to absence of infection.

Impairment of certain components of the RNAi pathway by injection of dsRNA resulted in significant effects (P<0.05) on time of appearance and titer of infectious virus in mosquito saliva. On days 10 and 12 post infection (Fig 5B and 5C), the infectious virus titers in feeding solutions from at least one of the mosquito groups with impaired RNAi (dsRNA.dcr2-, dsRNA.ago2-, and dsRNA.r2d2-injected) were higher (P<0.05) than in control groups (dsRNA.βGAL-injected, PBS-injected, and non-injected). At day 7, the titers in feeding solutions were not significantly different (Fig. 5A). At day 10, feeding solutions collected from dsRNA.dcr2-injected mosquitoes had significantly higher titers (P<0.05) than control groups (dsRNA.βGAL-injected, PBS-injected, and non-injected) (Fig. 5B). At day 12 the feeding solution titers from dsRNA.dcr2- and dsRNA.ago2-injected mosquitoes were significantly higher (P<0.05) than from all control groups, and dsRNA.r2d2-injected mosquito saliva titer was higher (P<0.05) than the non-injected group (Fig. 5C). Analysis by Chi-square test revealed that dsRNA.dcr2-injected mosquitoes had a significantly higher (P = 0.053) number of infected feeding solutions compared to the non-injected group at 7 dpi (Fig. 5A). At 10 dpi dsRNA.r2d2- and dsRNA.dcr2-injected groups had increased proportions (P<0.05) of feeding solutions infected compared to the non-injected group (Fig. 5B). At 12 dpi, dsRNA.ago2-, dsRNA.r2d2- and dsRNA.dcr2-injected groups had higher proportions (P<0.05) of feeding solutions infected (Fig. 5C).

## Discussion

We previously have identified a number of putative RNAi genes in *An. gambiae* and *Ae. aegypti* genome databases, including *dcr2*, *r2d2* and *ago2*, and by limited functional assays have confirmed the role of *dcr2* and *ago2* in RNAi [23]–[27]; (Franz and Olson, unpublished data). We also have demonstrated that the RNAi pathway in *Ae. aegypti* inhibits the replication of alphaviruses and flaviviruses if first triggered by introducing dsRNA derived from virus genome sequences [23],[26],[27]. We show here that RNAi is implemented naturally after acquisition of DENV2 by mosquitoes via the normal, oral route of infection. DENV2 replication generated virus-specific small RNAs, consistent in size with siRNAs, in mosquito cell cultures as well as tissues of female mosquitoes. However, despite the presence of siRNAs in their tissues, DENV2-competent mosquitoes accumulated viral genome RNA and infectious virus in midguts and other tissues and transmitted virus in saliva. We found that RNAi-mediated knock-down of *Aa-r2d2*, as well as *Aa-ago2* and *Aa-dcr2*, resulted in increased virus replication and shortened EIP in *Ae. aegypti*.

Cultured *Ae. aegypti* cells became persistently infected and released increasing titers of infectious virus until at least 10 dpi. DENV2-competent mosquitoes accumulated viral RNA and infectious virus in the midgut up to 10 dpi. The decline in virus titer and E antigen in the midgut after 10 dpi may have been a result of clearance of virus by defense mechanisms mediated by RNAi as well as the Toll pathway [8] or due to the nutritional status of the mosquito, since these mosquitoes were not given multiple blood meals; however, we found that the DENV2 infection persisted in tissues outside the midgut, including salivary glands, to at least 21 dpi, which is consistent with previous findings [5].

DENV2 infections generated substantial amounts of dsRNA in infected Aag2 cells and midguts of *Ae. aegypti* mosquitoes. Others have shown by IFA that dsRNA generated in DENV2-infected mammalian cells co-localizes with non-structural viral proteins known to be part of the replication complex within double-membrane vesicles [35]. The fact that we detected siRNA-like RNAs by northern blot hybridization using both sense and antisense probes suggests that dsRNAs associated with DENV2 replication are vulnerable to cleavage by Dcr-2 protein. This was particularly true in Aag2 cells, where we detected antisense siRNAs that undoubtedly resulted from processing of dsRNA that was a part of a replication intermediate. However, in contrast to mosquito cells where RNAi is induced by expression of a dsRNA transcript and siRNA polarity appears to be symmetrical [23],[26], in infected Aag2 cells we observed a preponderance of DENV2-specific siRNAs arising from the genome strand, suggesting that intramolecular secondary structure of viral genomic RNA may account for much of the dsRNA cleaved by Dcr2 to produce siRNAs. Others have shown similar asymmetry in siRNA polarity and have hypothesized that secondary structure of genomic RNA for some positive strand RNA viruses of plants and animals contributes substantially to siRNA formation [36],[37]. The sequences of the DENV2-specific siRNAs will be analyzed to accurately describe the population of 20–26 nt RNAs in DENV2 infected mosquito cells.

To show that the RNAi pathway affects virus replication in a competent DENV2 vector, we transiently silenced key RNAi pathway genes and examined resulting changes in virus titer. Previously, we have shown that injection of dsRNA derived from *An. gambiae ago2* gene sequence transiently impaired the RNAi pathway and significantly increased replication of O'nyong-nyong virus (genus, *Alphavirus*; family *Togaviridae*) in the vector [25]. We suggested that a robust RNAi response normally prevented widespread infection by ONNV in *An. gambiae*. Injection of dsRNAs targeting *Aa-dcr2* and *Aa-ago2* mRNAs reversed RNAi-based DENV2 resistance in the Carb77 transgenic line [23] (Franz and Olson, unpublished data). In the current study, we intrathoracically injected HWE mosquitoes with dsRNA.dcr2, dsRNA.ago2 or dsRNA.r2d2 prior to DENV2 infection and showed reduced corresponding mRNA 2 days later, at the time of infection. We observed that reduced expression of key RNAi genes resulted in increased mean virus titers in mosquitoes at 7 dpi. Injection of dsRNA.dcr2 was most effective for mRNA knock-down, and targeting *Aa-dcr2* by dsRNA.dcr2 injection caused the largest increase in infection rate and virus titers. The average titer for mosquitoes injected with dsRNA.dcr2 was >10-fold higher than the average titer of control dsRNA-treated mosquitoes and a small but significant number of dsRNA.dcr2- and dsRNA.ago2-injected mosquitoes had virus titers 100-fold higher than in control infections. If expression of RNAi component genes and activity of the RNAi pathway is variable among vector populations this may explain in part observed differences in vector competence [38]. We are currently determining whether there are genetic associations between RNAi genes such as *Aa-dcr2* and vector competence [39].

Finally, given the increase in DENV2 titers in dsRNA.dcr2-, dsRNA.r2d2- and dsRNA.ago2-treated mosquitoes, we asked whether the EIP is affected in mosquitoes with an impaired RNAi pathway. In mosquitoes pre-treated with dsRNA.dcr2, DENV2 transmission was detected as early as 7 dpi, indicating a shorter EIP when this defense mechanism was diminished. This could have significant epidemiological implications for DENV2 transmission, affecting vectorial capacity, which includes vector competence, but also incorporates all the other biological, behavioral, and ecological attributes of the vector that function in virus transmission. If the adult female mosquito has a shorter EIP and transmits virus more quickly after infection, the mosquito will have more opportunities to transmit virus during its lifetime.

An important function of RNAi in the mosquito may be to place limits on RNA virus replication and in the absence of RNAi, DENV2 and other arboviruses may increase their amplification to levels that begin to compromise vector fitness. This situation has been observed in *Drosophila* with *dcr2* or a*go*2 loss-of-function mutations that are highly vulnerable to RNA virus infections due to disabled RNAi responses [10]. Although we believe that the early innate immune response is important in limiting virus replication in mosquitoes, injection of dsRNA results in a transient impairment of the RNAi defense system and longer-term suppression induced by a genomic mutation might cause a more pronounced and effective response. Future work should lead toward understanding natural variations in expression of RNAi pathway genes in vector populations and resulting effects on DENV infection and transmission. We are currently developing null-mutants for *dcr2* in *Ae. aegypti* to more fully understand the role of this RNAi pathway gene in vector competence and vectorial capacity, and determine the possible effect of RNAi on infection of the vector by various DENV2 genotypes.

Variations in interaction of DENVs with the RNAi pathway could also have implications for determining why certain genotypes of DENVs are more efficiently transmitted than other genotypes and help explain the rapid spread of potentially virulent genotypes in the Americas [40],[41]. We hypothesize that DENV2 genotypes that most effectively circumvent RNAi would more efficiently replicate and be transmitted. An important question is how do arboviruses like DENV2 evade RNAi? Pathogenic RNA viruses of insects such as drosophila C virus express RNAi suppressors that increase viral pathogenicity, thus facilitating virus transmission to other insects [10],[42]. However, for efficient transmission of arboviruses to vertebrate hosts during acquisition of blood meals by the mosquito, it may be advantageous for the virus not to harm the vector. Indeed, the balance between RNAi activity in the vector and viral circumvention mechanisms might be a determinant in maintenance of the persistent virus infection. The La Crosse virus (LACV; *Bunyaviridae*) nonstructural protein NSs was reported to suppress RNAi in mammalian cells [43], but other studies have demonstrated that in mosquito cells persistently infected with recombinant LACV, expression of functional NSs protein has no effect on specific RNAi responses [44]. Another mechanism arboviruses may use to evade RNAi is minimizing early detection of the dsRNA trigger by the RNAi machinery. Geiss et al. [31] showed a significant reduction in WNV RNA in mammalian cells that were pre-treated with virus-specific siRNAs; however, cells that were treated subsequent to the establishment of viral replication did not have the same reduction in viral mRNA, suggesting that replicating viral RNA may be sequestered from the RNAi machinery in the cell. Identifying possible mechanisms of RNAi suppression or evasion associated with DENV2 infections is now being pursued.

Others have recently suggested that West Nile virus (WNV; *Flaviviridae*) fails to trigger RNAi in cultured C6/36 (*Ae. albopictus*) mosquito cells; however, the RNAi response in *Drosophila* appears to be protective against WNV infection [45]. We show here that the RNAi pathway in *Ae. aegypti* is not completely protective against DENV2 infection but modulates replication, suggesting that DENV2 has co-adapted to the major vector's RNAi response in ways as yet undefined. We hypothesize that RNAi is critical to maintaining a persistent virus infection in the vector, leading to long-term survival of the infected mosquito and efficient transmission of the virus with each successive blood meal. Important goals of future studies will be to determine how the virus mitigates the effects of RNAi in the vector, whether RNAi interacts with other innate defenses such as Toll pathway responses, and the consequences of impaired RNAi on vector fitness following infection with arboviruses.

## Materials and Methods

### Virus and cell culture

LLC-MK2 monkey kidney cells and C6/36 (*Ae. albopictus*) cells were cultured in modified Eagle's medium (MEM) supplemented with 8% fetal bovine serum, L-glutamine, non-essential amino acids and penicillin/streptomycin and maintained at 37°C and 28°C, respectively. Aag2 (*Ae. aegypti*) cells were cultured in Schneider's Drosophila medium with 10% fetal bovine serum, L-glutamine, non-essential amino acids and penicillin/streptomycin and maintained at 28°C. High passage DENV2 (Jamaica 1409) was used to infect fresh cultures of C6/36 cells to prepare infectious blood meals. Briefly, monolayers of C6/36 cells were inoculated with DENV2 at a MOI of 0.01 and held at 28°C; 6 days later medium was replaced and infected cells and medium were harvested at 12–14 days.

### Infectious virus titration by plaque assay

LLC-MK2 cells were grown to confluent monolayers in 24-well plates, infected with 10-fold serial dilutions of virus for 1 hour, then overlaid with an agarose-nutrient mixture. After 7 days incubation at 37°C cells were stained with 5 mg/ml MTT (3-[4,5-dimethylthiazol-2-yl]-2,5-diphenyltetrazolium bromide) solution and incubated for 4 hours [46],[47]. Viral titers were determined by counting plaques. Individual mosquitoes were triturated in 1.0 ml of L15 medium to release infectious virus as previously described [23]. Individual mosquito titers are reported as plaque forming units (pfu) per ml (values are expressed as the means±SEM).

### Mosquito rearing and infection with arboviruses

*Ae. aegypti* HWE mosquito eggs were hatched from an egg liner (containing 10,000–100,000 eggs) in 150 ml of deionized, autoclaved water. Larvae were transferred to a large rearing pan, collected as pupae 7–9 days later, transferred to an emergence container within a cage and maintained in the insectary at 28°C, 82% relative humidity until adult mosquitoes were harvested. Groups of 200 one-week-old adult females were placed in 2.5 liter cartons, deprived of sugar source overnight and allowed to feed on artificial blood meals consisting of virus-infected C6/36 cell suspension (60% vol/vol), 40% (vol/vol) defibrinated sheep blood (Colorado Serum Co., Boulder, CO) and 1 mM ATP. Virus titers were usually 10^6^–10^7^ pfu/ml for DENV2 [48]. The artificial blood meal was prewarmed to 37°C and then pipetted into water-jacketed glass feeders covered with a hog gut membrane and maintained at a constant temperature of 37°C. Feeders were placed onto the net covering the cartons to allow females to feed through the hog gut membrane for 1 h. Fed females were selected, put into new cartons, provided with water and sugar and maintained in the insectary for analysis.

### Northern blot analysis of mRNA

Viral RNA and mRNA originating from endogenous mosquito genes were analyzed by northern blot hybridization performed as described [49]. Briefly, total RNA was extracted from mock or DENV2 infected Aag2 cells (MOI = 0.05) or whole mosquitoes using Trizol reagent (Invitrogen) following the manufacturer's instructions; 3–5 µg of total RNA were electrophoresed in a 1.2% agarose gel and blotted onto a positively charged nylon membrane (Ambion). Blots were hybridized with antisense ^32^P-UTP-labeled RNA probes that were transcribed *in vitro* from linearized pBluescript II SK (Stratagene) containing a cDNA insert derived from the specific sequence of DENV-2 (prM), *Aa-dcr2*, *Aa-r2d2* or *Aa-ago2* RNA., and hybridization was visualized using a phosphorimager. Alternatively, random-primed ^32^P-dCTP-labeled DNA probes were generated from the same template by using the Megaprime DNA Labeling Kit (Amersham Pharmacia Biosciences).

### Northern blot analysis of siRNA

Approximately 50 µg of total RNA obtained from mock or DENV2 infected Aag2 cells (Fig 1D) or 50 µg of total RNA from 60 mosquitoes (Fig 3C) were loaded per lane onto a 15% polyacrylamide-TBE-urea denaturing gel and separated by electrophoresis. RNA was electrophoretically transferred to a non-charged nylon membrane and chemically cross-linked to the membrane using carbodiimide [50]. The membrane was incubated in UltraHyb (Ambion) hybridization buffer at 42°C for 30 minutes. For detecting siRNAs from infected Aag2 cells or mosquitoes, single-stranded RNA probes were transcribed in sense or antisense orientation from the 498 bp cDNA encoding the DENV2 prM gene using the MEGAscript kit (Ambion), with approximately 9% of the UTP in the transcription reaction conjugated to biotin for detecting siRNA from Aag2 or with ^32^P-UTP for detecting siRNA from mosquitoes. Five micrograms of labeled sense or antisense RNA were hydrolyzed to 50–100 nt fragments in 200 mM carbonate buffer at 60°C for approximately 2.5 hours. Probe was added to the UltraHyb buffer after the pre-hybridization and incubated for 16 hr at 42°C. The membrane was washed twice with 2× SSC-0.1% SDS for 5 minutes each, followed by two washes in 0.1× SSC-0.1% SDS for 15 minutes each. Biotin-labeled probes were detected with the BrightStar BioDetect Kit (Ambion) and membranes were exposed to X-ray film for 18 hours (Fig. 1D), and ^32^P-labeled probes were detected using a phosphorimager (Fig. 3C).

### Detection of siRNAs derived from DENV2 RNA in mosquito midguts

siRNAs were enriched from 200 µg total midgut RNA extracted from female mosquitoes 1–14 days post bloodmeal according to a previously described protocol [23]. High molecular-weight RNA was precipitated by addition of 5% (wt/vol) polyethylene glycol (*M*r 8,000) and 0.5 M NaCl. Supernatants containing siRNAs were analyzed in a ribonuclease protection assay by using the *mir*Vana miRNA Detection Kit (Ambion). For nuclease protection, siRNAs were hybridized with an antisense RNA probe 30 nt in length containing 22 nt of sequence complementary to a portion of the prM-encoding region of DENV2 RNA (Integrated DNA Technologies, Colorado State University). RNA probes were end-labeled with [γ-^32^P] ATP. Hybridizations were performed at 42°C. After RNase digestion, hybridized RNA samples were electrophoretically separated on a 16% polyacrylamide gel containing 7 M urea.

### Detection of dsRNA in Aag2 cells and midguts using J2 antibody

Aag2 cells were infected with DENV2 at a MOI of 0.1 and *Ae. aegypti* mosquitoes were given a non-infectious or infectious blood meal containing ∼10^6^ pfu/ml of virus. Both cells and midguts were fixed in 4% paraformaldehyde in PBS at 4–5 dpi and 7 dpi, respectively. dsRNA was detected using J2 antibody (Scicons, Hungary) as previously described with minor modifications [33]. All images were taken with a Leica DM 4500B microscope.

### Generation of dsRNA and delivery to mosquitoes

Oligonucleotide primers incorporating T7 RNA polymerase promoter sequences at the 5′ ends were designed to amplify ∼500-bp regions of RNAi pathway genes (Table 1) to serve as transcription templates for dsRNAs for *Aa-dcr2*, *Aa-r2d2* and *Aa-ago2* according to the method previously described [25]. One week old adult female mosquitoes were intrathoracically injected with 500 ng of each dsRNA prior to infection.

### Analysis of virus transmission potential

Groups of 200 *Ae. aegypti* HWE mosquitoes were intrathoracically injected with dsRNA.r2d2, dsRNA.ago2, dsRNA.dcr2, dsRNA.βGAL (500 ng dsRNA/mosquito), or PBS. A separate group of mosquitoes was not injected. Two days later all mosquito groups were challenged with an infectious blood meal containing 6.6×10^6^ pfu/ml of DENV2. At pre-determined times, groups of 10 mosquitoes were allowed to probe and feed on 350 µl of a feeding solution (50% FBS/164 mM NaCl/100 mM NaHCO_3_/0.2 mM ATP/∼50 µg sucrose/phenol red, pH 7) that was placed between two parafilm membranes stretched over a glass feeder. After probing, mosquitoes and feeding solutions were collected and prepared for plaque assays [23].

### Statistical analysis

To determine if impairment of the RNAi pathway in mosquitoes has an effect on virus replication and EIP, data were subjected to analysis of variance (ANOVA) using the general linear model of SAS (SAS User's Guide, Cary, NC: Statistical Analysis System Institute, Inc., 1987). Sources of variation for virus titer in mosquitoes were treatment (non-injected, dsRNA.βGAL-, dsRNA.Aa.ago2-, dsRNA.Aa.r2d2-, and dsRNA.Aa.dcr2-injected) and replicate (4); and for feeding solution titer were treatment (non-injected, PBS-, dsRNA.βGAL-, dsRNA.Aa.ago2-, dsRNA.Aa.r2d2-, and dsRNA.Aa.dcr2-injected), replicate (2), days post-infection (7, 10, and 12), and the effect of treatment by day post infection. Since variation between replicates was not significant, it was removed from the model. If variances were not homogeneous, data were subjected to log 10 transformations. When differences among treatment means were detected, they were separated using least significant difference (LSD) procedure. The effect of treatments on the number of mosquitoes infected and on the number of feeding solutions infected was analyzed by chi-square test (SAS). For the number of mosquitoes infected each experimental group was compared to the non-injected group; for the number of feeding solutions infected each experimental group was compared to the average of the three control groups.
